# A Conserved Non-Canonical Docking Mechanism Regulates the Binding of Dual Specificity Phosphatases to Cell Integrity Mitogen-Activated Protein Kinases (MAPKs) in Budding and Fission Yeasts

**DOI:** 10.1371/journal.pone.0085390

**Published:** 2014-01-22

**Authors:** Almudena Sacristán-Reviriego, Marisa Madrid, José Cansado, Humberto Martín, María Molina

**Affiliations:** 1 Departamento de Microbiología II, Facultad de Farmacia, Universidad Complutense de Madrid, and Instituto Ramón y Cajal de Investigaciones Sanitarias (IRYCIS), Madrid, Spain; 2 Yeast Physiology Group, Departamento de Genética y Microbiología, Facultad de Biología, Universidad de Murcia, Murcia, Spain; Instituto de Salud Carlos III, Spain

## Abstract

Dual-specificity MAPK phosphatases (MKPs) are essential for the negative regulation of MAPK pathways. Similar to other MAPK-interacting proteins, most MKPs bind MAPKs through specific docking domains known as D-motifs. However, we found that the *Saccharomyces cerevisiae* MKP Msg5 binds the MAPK Slt2 within the cell wall integrity (CWI) pathway through a distinct motif (IYT). Here, we demonstrate that the IYT motif mediates binding of the Msg5 paralogue Sdp1 to Slt2 as well as of the MKP Pmp1 to its CWI MAPK counterpart Pmk1 in the evolutionarily distant yeast *Schizosaccharomyces pombe*. As a consequence, removal of the IYT site in Msg5, Sdp1 and Pmp1 reduces MAPK trapping caused by the overexpression of catalytically inactive versions of these phosphatases. Accordingly, an intact IYT site is necessary for inactive Sdp1 to prevent nuclear accumulation of Slt2. We also show that both Ile and Tyr but not Thr are essential for the functionality of the IYT motif. These results provide mechanistic insight into MKP-MAPK interplay and stress the relevance of this conserved non-canonical docking site in the regulation of the CWI pathway in fungi.

## Introduction

Among the most ancient signalling routes are the mitogen-activated protein kinase (MAPK) pathways that transduce extracellular stimuli into appropriate cellular responses. MAPKs contain a highly conserved TXY motif in the kinase activation loop in which both Thr and Tyr residues must be phosphorylated to achieve kinase activation [1]. Their main negative regulators are therefore protein phosphatases. Prominent among these is a subfamily of dual-specificity phosphatases (DSPs) that specifically dephosphorylate both Thr and Tyr residues in target MAPKs. These enzymes are designated as dual-specificity MAPK phosphatases (MKPs) and play an essential role in the regulation of MAPK signalling [2], [3]. This MKP-mediated dephosphorylation event not only requires a transient enzyme-substrate interaction involving the active site of these enzymes, but also the formation of a complex through specific docking interactions between conserved regions within both MKPs and MAPKs [4]. Such docking sites are located outside the catalytic domains of these proteins and promote binding specificity and high affinity interactions. One of the best characterised docking motifs found in MAPK phosphatases and other MAPK-interacting proteins is the D-domain, which is also known as the kinase interaction motif (KIM). D-domains comprise a cluster of basic residues followed by a hydrophobic sub-motif containing Leu, Ile or Val separated by one residue (Lys/Arg_1–3_-X_2–6_-φ-X-φ) [5],[6]. A second type of MAPK-docking site named the DEF motif (for docking site for ERK FXFP) is also present in the MAPK phosphatase MKP-1 [7]. Whereas D-motifs interact with acidic residues in the common docking (CD) domain of MAPKs, DEF motifs bind to a hydrophobic pocket that is only exposed upon MAPK activation [8].

All eukaryotic cells possess multiple MAPK pathways, which coordinately regulate many physiological processes. *Saccharomyces cerevisiae* has five MAPK signalling pathways involved in mating, pseudohyphal/invasive growth, osmoregulation, ascospore formation and cell wall integrity (CWI) [9]. The CWI pathway is mediated by the MAPK Slt2 and is responsible for orchestrating changes in the cell wall through the cell cycle and in response to various forms of stress, especially cell wall insults, to ensure cell survival [10]. Therefore, this pathway is tremendously important for yeast physiology, and consequently it is conserved among fungal species [11]. Then, MAPKs orthologous to *S. cerevisiae* Slt2 operate in other fungi, like Pmk1 in *Schizosaccharomyces pombe*. Not only MAPKs but many other components participating in fungal CWI pathways are conserved [11]. MKPs orthologous to *S. cerevisiae* Msg5 and its paralogue Sdp1, both of which dephosphorylate the MAPK Slt2 [12]–[14], are also broadly present in other fungi. In *S. pombe*, the MKP Pmp1 is responsible for dephosphorylating the CWI MAPK Pmk1 [15].

The MKP Msg5 is not only regulating the CWI but also the mating pathway in *S. cerevisiae*. We have recently shown that, whereas the interaction with the CD domains of the mating MAPKs Fus3 and Kss1 involves a canonical Msg5 D-domain, a novel motif (I^102^Y^103^T^104^) in Msg5 is mediating binding to the CWI MAPK Slt2 and its pseudokinase paralogue Mlp1 [16]. To build on these findings and broaden the work beyond *S. cerevisiae* Msg5, we have studied the relevance of this motif for other yeast MKPs. Here we show that the IYT site is also involved in the interaction of Sdp1 to Slt2, and in Pmp1 binding to its partner MAPK Pmk1 in *S. pombe*, highlighting the importance of this motif in the interaction of MKPs with fungal CWI MAPKs.

## Materials and Methods

### Yeast strains and culture conditions

The *S. cerevisiae* strains used in this work were DD1-2D (*MAT* **a** *ura3 his2 trp1 leu2 ade1 msg5-1*Δ*::LEU2*) [17], YPH499 (*MAT* **a**, *ade2-101 trp1-63 leu2-1 ura3-52 his3-Δ200 lys2-801*), BY4741 (*MAT* **a**, *his3Δ1 leu2Δ met15Δ0 ura3Δ0*) and Y00897 (BY4741 isogenic, *gas1Δ::Kan*MX4) from Euroscarf (Frankfurt, Germany). Yeast cultures were performed as reported previously [18]. When necessary, Congo red (Sigma) was added at the indicated concentrations.

The *S. pombe* strains employed were MI200 (*h^+^ pmk1-HA6H:ura4^+^*) [19], MI102 (*h^+^ pmk1::KanR*) and MI212 (*h^+^ pmp1::KanR pmk1-HA6H:ura4^+^*) [20], DP400 (*h^+^ pmp1::KanR pmp1-GST:leu1^+^ pmk1-HA6H:ura4^+^*), DP410 (*h^+^ pmp1::KanR pmp1(C158S)-GST:leu1^+^pmk1-HA6H:ura4^+^*), DP420 (*h^+^ pmp1::KanR pmp1(I10A Y11A T12A)-GST:leu1^+^ pmk1-HA6H:ura4^+^*), and DP430 (*h^+^ pmp1::KanR pmp1(I10A Y11A T12A C158S)-GST:leu1^+^ pmk1-HA6H:ura4^+^*) (this work). All strains are *ade6-M216 leu1-32 ura4-D18*. They were grown at 28°C in rich medium YES with 2% glucose, and supplemented with adenine, leucine, histidine or uracil (100 mg/litre, Sigma Chemical), depending on specific requirements [19].

### DNA manipulation and plasmids

General DNA methods were performed using standard techniques. In order to express Mlp1 fused to GST in *S. cerevisiae* cells, pEG(KG)-Mlp1 was constructed by PCR amplifying the corresponding *MLP1* region using pET15b-Mlp1 [16] as a template and the primers FMLP1 5- TCGCGGATCCATGGCGACTGACACCGAGAG-3 (*Bam*HI site is underlined) and 5- TCGCGTCGACTTAGTTAACACCCTGAAATGAATTAGAG-3 (*Sal*I site is underlined). The *Bam*HI-*Sal*I digested PCR product was sub-cloned into pEG-KG (2 µm, *GAL1–10UAS-CYC1*) [21].

To express Sdp1-Myc in *S. cerevisiae*, the plasmid YCplac22Sdp1m was constructed. To this end, primers PROSdp1 5-CATGAAGCTTGGTGGCCCAAAGTAACACTTC-3 (*Hind*III site is underlined) and RSdp1 5-TCGCGGATCCCGGTACTTTTCTATAACTGTTGGC-3 (*Bam*HI site is underlined) were used to amplify a fragment including the complete ORF and starting 1 Kb upstream of the initiation codon of *SDP1*. This fragment was cloned into pGEM-T to obtain pGEMT-PSDP1. The *Hind*III-*Bam*HI fragment was inserted into pRS305myc [12] to obtain pRS305SDP1m. Then, the *Hind*III-*Sac*I DNA fragment from pRS305SDP1m carrying the *MYC_6_* epitope was sub-cloned into YCplac22 (*CEN4*, *TRP1*) to give YCplac22SDP1m. The YCplac22Sdp1^IAYATA^m plasmid was generated by site-directed mutagenesis of YCplac22SDP1m with the oligonucleotides FSDP1D 5-GTAGCCATAATCTCGCATGAACGCAGCTGCATCACCCACGCGAACACCGAAC-3 and RSDP1D 5- GTTCGGTGTTCGCGTGGGTGATGCAGCTGCGTTCATGCGAGATTATGGCTAC-3.

To overexpress Sdp1 and Sdp1^IAYATA^, the *SDP1* ORF was PCR-amplified with primers FYSDP1 5-CATGAAGCTTCTTGTAGCCATAATCTCGCATG-3 (*Hind*III site is underlined) and RYSDP1 5-TCGCGAGCTCACTAGGTAATACGACTCACTATAG-3 (*Sac*I site is underlined) using YCplac22Sdp1m and YCplac22Sdp1^IAYATA^m plasmids as templates and then the *Hind*III-*Sac*I digested fragments were sub-cloned into pYES2 (2μ *URA3 GAL1*) (Invitrogen). pYES2SDP1^C140A^myc and pYES2SDP1^C140A-IAYATA^myc plasmids were generated by site-directed mutagenesis of pYES2SDP1myc and pYES2SDP1^IAYATA^myc, respectively, following the method previously described [12] using the oligonucleotides FSDP1C 5- GAAGATACTGATACATGCCCAATGTGGGCTCTCGAGATCCGCAACG-3 and RDP1C 5- CGTTGCGGATCTCGAGAGCCCACATTGGGCATGTATCAGTATCTTC-3. pYES2SDP1^IA^myc, pYES2SDP1^YA^myc and pYES2SDP1^TA^myc were obtained amplifying the *SDP1* ORF with the reverse primer RYSDP1 and the following forward primers FYSDP1^IA^ 5-AAGCTTCTTGTAGCCATAATCTCGCATGAACGCATAC-3, FYSDP1^YA^ 5-CATGAAGCTTCTTGTAGCCATAATCTCGCATGAACGCATACACATCACCCACG-3 and FYSDP1^TA^ 5-AAGCTTCTTGTAGCCATAATCTCGCATGAACATATACGCATC-3 (*Hind*III sites are underlined), respectively, using pYES2SDP1myc as template. The *Hind*III-*Sac*I digested fragments were sub-cloned into pYES2.

Recombinant Sdp1-GST and Sdp1^IAYATA^-GST were obtained using pETGEXCT plasmid [22]. To this end, *SDP1* and *SDP1^IAYATA^* were amplified with upstream primers 5- CATGCCATGGCCATGAACATATACACATCACCCACG-3 or 5-CATGCCATGGCCATGAACGCAGCCGCATCACC-3, respectively, (*Nco*I sites are underlined) and the downstream primer 5-TCGCGAGCTCCGGTACTTTTCTATAACTGTTGGC-3 (*Sac*I site is underlined). PCR products were sub-cloned into pGEM-T, and then the *Nco*I-*Sac*I fragments carrying *SDP1* and SDP1^IAYATA^ from the generated plasmids were sub-cloned into *Nco*I-*Sac*I sites of pETGEXCT. To produce recombinant Slt2 and Mlp1 His-tagged proteins, pET15B-derived plasmids pET15B-Slt2-His and pET15B-Mlp1-His were used as described previously [16].

The YEp352GST plasmid was obtained by cloning the GST-encoding *Eco*RI-*Sac*I fragment from pEG(KG) into YEp352 [23]. Plasmids YEp352MSG5GST and YEp352MSG5^C319A^GST expressing Msg5-GST and Msg5^C319A^-GST, respectively, were generated in two steps. Firstly, the *Bam*HI fragment carrying the promoter and the corresponding ORFs of *MSG5* from YCplac22MSG5m or YCplac22MSG5^C319A^m [12] were sub-cloned into YEp352. Secondly, the *Sma*I fragment from YEp352GST-bearing GST was sequentially cloned at the *Sma*I site. The YEp352MSG5^C319A-IAYATA^GST plasmid was obtained by site-directed mutagenesis on YEp352MSG5^C319A^GST using the mutagenic primers 5′-AGCGAGGCCTCTGCAGCTGCACTACCAACATCTTTGAAGAACCGAACTG-3′ and 5′-AGATGTTGGTAGTGCAGCTGCAGAGGCCTCGCTTCGCCTCATCGACAGC-3′.

In order to determine *MLP1* transcriptional induction, the episomic vector YEp352, bearing an *MLP1-GFP* fusion (pMLP1-GFP) [24], or pMLP1-LacZ, carrying the transcriptional fusion of the *MLP1* promoter to the *lacZ* gene [25], were used. For Slt2 localisation studies, cells were transformed with the plasmid pRS425-*SLT2*-GFP [13].

To construct the integrative plasmid pIL-pmp1:GST, the *pmp1^+^* ORF plus regulatory sequences were amplified by PCR using *S. pombe* genomic DNA as a template, employing the 5′-oligonucleotide Ppmp1X-F (TATATTCTAGACTCCAGCATCACAGTCTTTTCCTTC), which hybridises at a position ∼700 bp upstream of the *pmp1^+^* ATG start codon and contains an *Xba*I site (underlined), and the 3′-oligonucleotide Ppmp1B-R (TATATGGATCCAGAAGCATCATTACTTATACTGCCGC), which hybridises at the 3′ end of *pmp1* ^+^ ORF (except the stop codon) and incorporates a *Bam*HI site. The resulting PCR fragment was digested with *Xba*I and *Bam*HI and cloned into the integrative plasmid pIL-GST [19].

The integrative plasmid pIL-pmp1(*C158S*):GST was obtained by site-directed mutagenesis employing pIL-pmp1:GST as template and the mutagenic oligonucleotides PpmpC158S-F (GTTTTAATTAATAGTCAAATGGGCA) and PpmpC158-R (TGCCCATTTGACTATTAATTAAAAC). To construct the plasmid pIL-pmp1(*I10A Y11A T12A*):GST, the mutagenic oligonucleotides used were PpmpAAA-F (ATGTCTCAAAAACTACCTCCCTTAAAAGCTGCCGCCTCCCAACTTCCCCTGGTTTC) and PpmpAAA-R (GAAACCAGGGGAAGTTGGGAGGCGGCAGCTTTTAAGGGAGGTAGTTTTTGAGACAT). The integrative plasmid pIL-pmp1(*I10A Y11A T12A C158S*):GST was prepared using pIL-pmp1(*I10A Y11A T12A*):GST as the template and the mutagenic oligonucleotides PpmpC158S-F and PpmpC158-R. In all cases, the mutated *pmp1^+^* ORFs plus regulatory sequences were amplified by PCR with the oligonucleotides Ppmp1X-F and Ppmp1B-R, digested with *Xba*I and *BamH*I, and cloned into pIL-GST, as above. Finally, the resulting plasmids were digested at the unique *Nru*I site within *leu1^+^*, and the linear fragments were transformed into strain MI212. Transformants *leu1^+^* were obtained and the correct integration of the fusions was verified by both PCR and Western blot analysis.

### Preparation of bacteria and yeast extracts

Recombinant GST- or His-tagged proteins were expressed in the *E. coli* strain Rosetta DE3 (Novagen). Cells were collected and lysed by sonication in PBS buffer containing 2 mM PMSF, 1 mM DTT, 1 mM EDTA, and 1 mg/ml Lysozyme in the presence of a protease-inhibitor cocktail (Roche). Extracts were clarified by centrifugation and then stored at −80°C. Budding and fission yeast extracts were obtained as previously described in [18] and [19], respectively.

### Binding assays

For *in vivo* binding assays, cells were collected and lysed as above in lysis buffer lacking SDS and NP40. Yeast lysates were incubated with glutathione-Sepharose beads (GE Healthcare) for two hours. Beads were washed extensively with the same buffer, resuspended in SDS loading buffer, and proteins were analysed by SDS-PAGE and immunoblotting as described [12]. *In vitro* binding assays were performed by mixing *E. coli* extracts containing GST or GST-fused proteins with *E. coli* extracts bearing the corresponding His-tagged proteins, and then processed as above.

Purification of Pmk1-HA6H and Pmp1-GST fusions was performed with Ni^2+^-NTA-agarose beads (Qiagen) and glutathione-Sepharose beads (GE Healthcare), respectively, as described previously [20].

### Immunoblotting analysis

In experiments carried out with budding yeast, immunodetection of Glucose-6-phosphate dehydrogenase (G6PDH) and Myc-tagged proteins was carried out using polyclonal anti-G6PDH (Sigma) and monoclonal 4A6 (Millipore) or 9E10 (Santa Cruz Biotechnology) antibodies, respectively. Polyclonal anti-phospho-p44/p42 MAPK (Thr202/Tyr204) (Cell Signaling), anti-GST (Santa Cruz Biotechnology) and anti-His antibodies (Sigma) were also used as described previously [18]. The primary antibodies were detected using a fluorescently-conjugated secondary antibody with an Odyssey Infrared Imaging System (LI-COR Biosciences).

In experiments performed with fission yeast, dual phosphorylation in Pmk1 was detected with polyclonal anti-phospho-p42/p44 as above, whereas total Pmk1 was detected after incubation with mouse monoclonal anti-HA antibody (12CA5, Roche Molecular Biochemicals).The immunoreactive bands were revealed with an anti-mouse-HRP-conjugated secondary antibody (Sigma) and the ECL system (GE Healthcare). GST fusions were detected with a goat anti-GST-HRP conjugated polyclonal antibody (GE Healthcare).

### *β-*Galactosidase activity assays

β-Galactosidase activity was determined according to [26]. Values are averages of at least three independent transformants assayed in duplicate.

### Flow cytometry assays

For Mlp1-GFP analysis, cells were collected, washed twice with PBS, and then analysed by flow cytometry in a Guava easyCyte flow cytometer, acquiring green fluorescence through a 488 excitation laser and a 525/30 BP emission filter (BFP). The marker was set using yeasts that do not express GFP.

### Fluorescence microscopy

For the visualisation of GFP on live yeast cells containing pRS425-*SLT2*-GFP, exponentially growing cultures were centrifuged at 3000 r.p.m., washed once with water and observed. Cells were examined under an eclipse TE2000U microscope (Nikon) and digital images were acquired with an Orca C4742-95-12ER charge-coupled-device camera (Hamamatsu Photonics) and Aquacosmos Imaging Systems software.

## Results

### The IYT motif of *S. cerevisiae* Sdp1 mediates binding to the CWI MAPK Slt2

**Figure 1:**
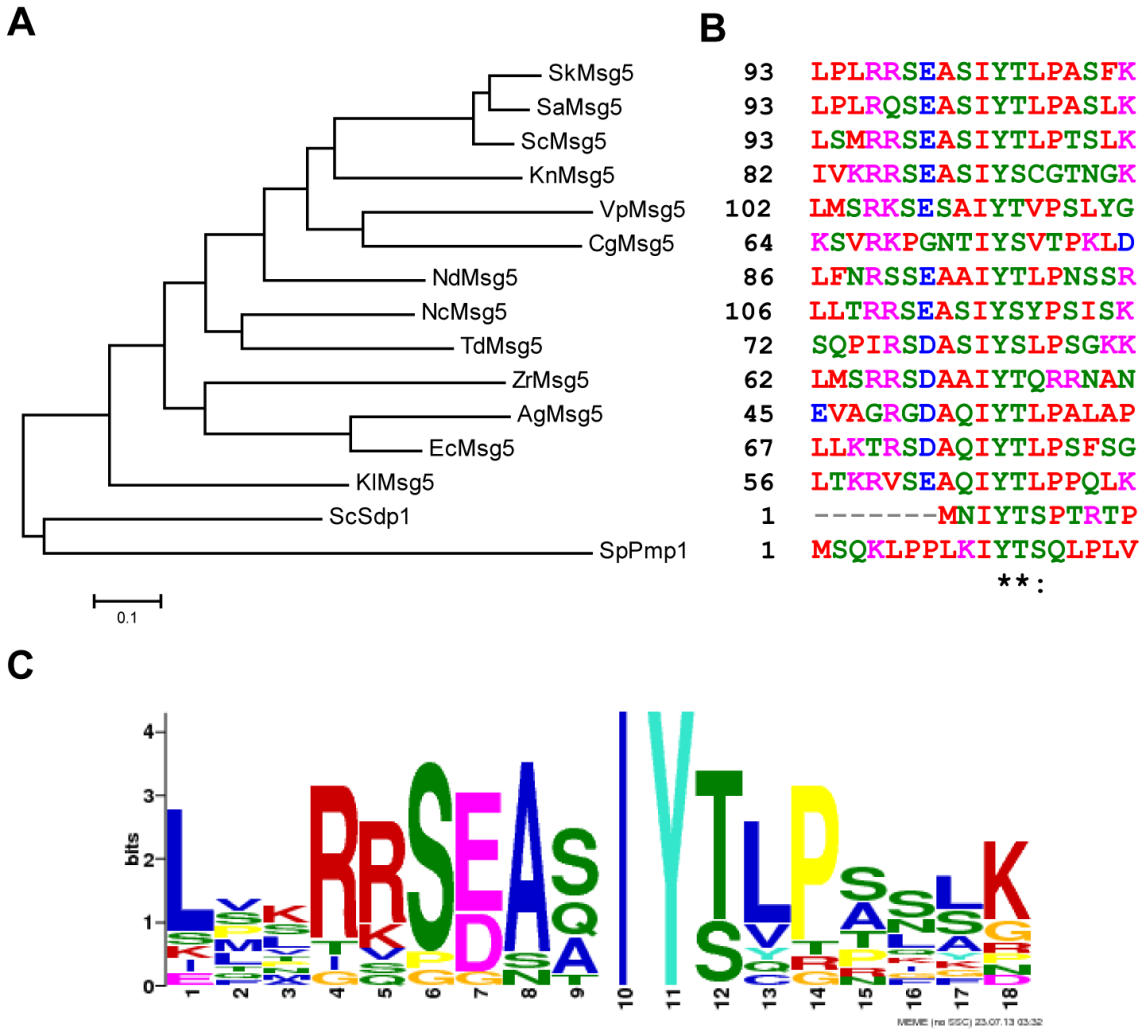
Conservation and consensus pattern of the IYT motif of yeast MKPs. (A) Dendrogram of *S. cerevisiae* (Sc) full sequences of genes encoding MKPs Msg5 and Sdp1 and the orthologous sequences from the indicated yeast species. (B) ClustalW multiple sequence alignment of the region containing the IYT motif within MKPs from *Saccharomyces kudriavzevii* (Sk), *Saccharomyces arboricola* (Sa), *Naumovozyma dairenensis* (Nd), *Naumovozyma castellii* (Nc), *Torulaspora delbrueckii* (Td), *Kazachstania naganishii* (Kn), *Vanderwaltozyma polyspora* (Vp), *Zygosaccharomyces rouxii* (Zr), *Ashbya gossypii* (Ag), *Kluyveromyces lactis* (Kl) *Candida glabrata* (Cg), *Schizosaccharomyces pombe* (Sp) and *Eremothecium cymbalariae* (Ec). Dendrogram was created with the software Mega 5.2. Conserved residues are dotted while identical residues are labelled with asterisks. (C) Consensus pattern and sequence logo of the IYT motif generated by the NEME software [38]. The overall height of each stack indicates the sequence conservation at that position (measured in bits), whereas the height of symbols within the stack reflects the relative frequency of the corresponding amino acid at that position.

Our BLAST search to know the conservation of the IYT motif among fungi showed the existence of a significant number of yeast Msg5 orthologues bearing this motif (Figure 1A). A detailed analysis of the amino acid sequence of the region surrounding this site in these proteins revealed some interesting features. For instance, the frequent presence of basic and acidic residues previous to the IYT signature, the invariant Ile and Tyr and the existence of either Thr or Ser as the third residue within the motif (Figures 1B and 1C). Among the MKPs carrying this motif are Sdp1 from *S. cerevisiae* and Pmp1 from the evolutionarily distant fission yeast *S. pombe* (Figure 1A). Interestingly, these two proteins are phylogenetically distant from *S. cerevisiae* Msg5, as observed in the dendrogram shown in Fig. 1A. We first addressed the functional conservation of this motif by analysing its importance in the interaction of Sdp1 with its target MAPK.

### Mutation of the IYT motif reduces the ability of wild type Sdp1 to down-regulate the CWI pathway and of inactive Sdp1 to act as a substrate trap for Slt2

Previous studies of cells lacking Sdp1 revealed no evident phenotype except for a slight increase of Slt2 phosphorylation in response to heat shock [13]. Our analysis of several *sdp1*Δ mutant strains from different cellular backgrounds showed that these mutants presented no sensitivity to cell wall perturbing agents and high temperature. Likewise, they also showed no alteration of Slt2 phosphorylation under these conditions in comparison to wild type cells (data not shown). Therefore, in order to analyse the relevance of the IYT motif for Sdp1 function, we studied the effect caused by the expression of high doses of Sdp1 and Sdp1^IAYATA^ in *S. cerevisiae*. To this end, we overproduced the Myc-tagged versions of the corresponding *SDP1* alleles from the *GAL1* promoter. Overexpression of a catalytically inactive form of Sdp1 has been shown to result in the accumulation of phosphorylated Slt2 [14]. Therefore, we also included in the study the phosphatase-dead version Sdp1^C140A^, which bears a mutation at the conserved cysteine that is known to abolish the catalytic activity within all PTPs, and the double mutant Sdp1^C140A-IAYATA^ that is also affected in the IYT site. To easily analyse their down-regulatory impact on CWI signalling, the overexpression experiments were carried out in a *gas1*Δ mutant strain, which presents a constitutively activated CWI pathway due to the lack of beta-1,3-glucanosyltransferase activity, which is required for cell wall assembly [27]. As readouts of the effect of the different Sdp1 versions on Slt2 function, we analysed both Slt2 phosphorylation and the transcriptional activity mediated by the main Slt2 substrate, the transcriptional factor *RLM1*. To this end, a reporter transcription assay based on the expression of *lacZ* under the control of the cell wall damage-inducible Rlm1-dependent *MLP1* promoter was used [25]. As can be seen in Figures 3A and 3B, the overexpression of Sdp1 considerably reduced the amount of phospho-Slt2 and the level of Rlm1-driven transcription in the *gas1* mutant. Mutation of the IYT motif within Sdp1 did not result in any apparent differences with wild type Sdp1 regarding the level of phosphorylated Slt2. In contrast, *MLP1* expression was significantly higher in comparison with that of cells expressing wild type Sdp1, indicating a reduced function of the mutant Sdp1^IAYATA^ version on Slt2 activity. As expected, expression of Sdp1^C140A^ caused an accumulation of phosphorylated Slt2 (Figure 3A), probably due to the formation of a stable complex of this inactive DSP form with Slt2, a phenomenon known as substrate trapping that occurs in other tyrosine phosphatases [28]. This stable association of inactive Sdp1^C140A^ with Slt2 seems to impede downstream signalling, since although leading to high phospho-Slt2 levels, a significant decrease in *MLP1*-driven beta-galactosidase activity was observed (Figure 3B). Of interest, removal of the IYT motif from catalytically inactive Sdp1 reduced the amount of phosphorylated Slt2 and increased Rlm1-dependent transcriptional activation (Figure 3, A and B), suggesting reduced binding of the double Sdp1^C140A-IAYATA^ mutant to Slt2. As seen in *gas1Δ* cells, this mutant version of Sdp1 also lost the ability to act as a substrate trap for Slt2 compared with Sdp1^C140A^ when overexpressed in wild type cells (Figure 3C).

**Figure 2:**
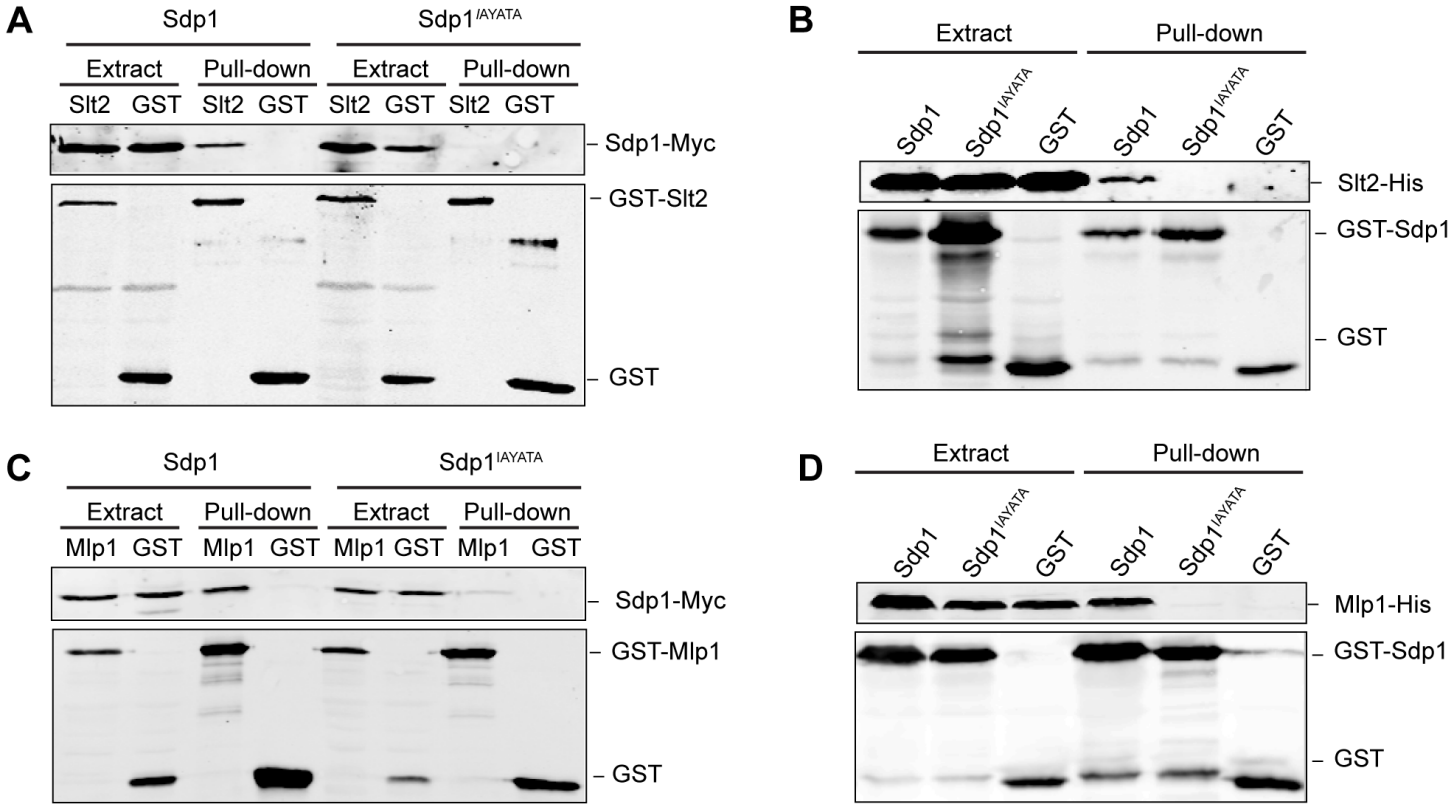
Involvement of the Sdp1 IYT motif on Slt2 and Mlp1 binding. (A) Western blotting analysis of *in vivo* co-purification of Sdp1 with Slt2. Cell extracts of the wild type strain YPH499 transformed with plasmids YCplac22Sdp1m (Sdp1) or YCplac22Sdp1^IAYATA^m (Sdp1^IAYATA^) and plasmids pEG(KG) (GST) or pEG(KG)-SLT2 (Slt2) were incubated with glutathione-Sepharose to pull-down GST-complexes. Immunodetection was performed with anti-Myc (upper panel) and anti-GST (lower panel) antibodies. (B) Western blotting analysis of *in vitro* co-purification of recombinant Sdp1 with recombinant Slt2. *E. coli* extracts containing GST, GST-Sdp1 or GST-Sdp1^IAYATA^ were mixed with *E. coli* extracts containing Slt2-His and then incubated with glutathione-Sepharose to pull-down GST-complexes. Immunodetection was performed using anti-polyHis (upper panel) and anti-GST (lower panel) antibodies. (C) Western blotting analysis of *in vivo* co-purification of Sdp1 with Mlp1. Cell extracts of the strain YPH499 transformed with plasmids YCplac22Sdp1m (Sdp1) or YCplac22Sdp1IAYATAm (Sdp1^IAYATA^) and plasmids pEG(KG) (GST) or pEG(KG)-MLP1 (Mlp1), were processed as in Figure 1B. (D) Western blotting analysis of *in vitro* co-purification of recombinant Sdp1 with recombinant Mlp1. Experiments were performed as in Figure 1C but using *E. coli* extracts containing Mlp1-His. Similar results were obtained in three different experiments and selected images correspond to representative blots.

**Figure 3:**
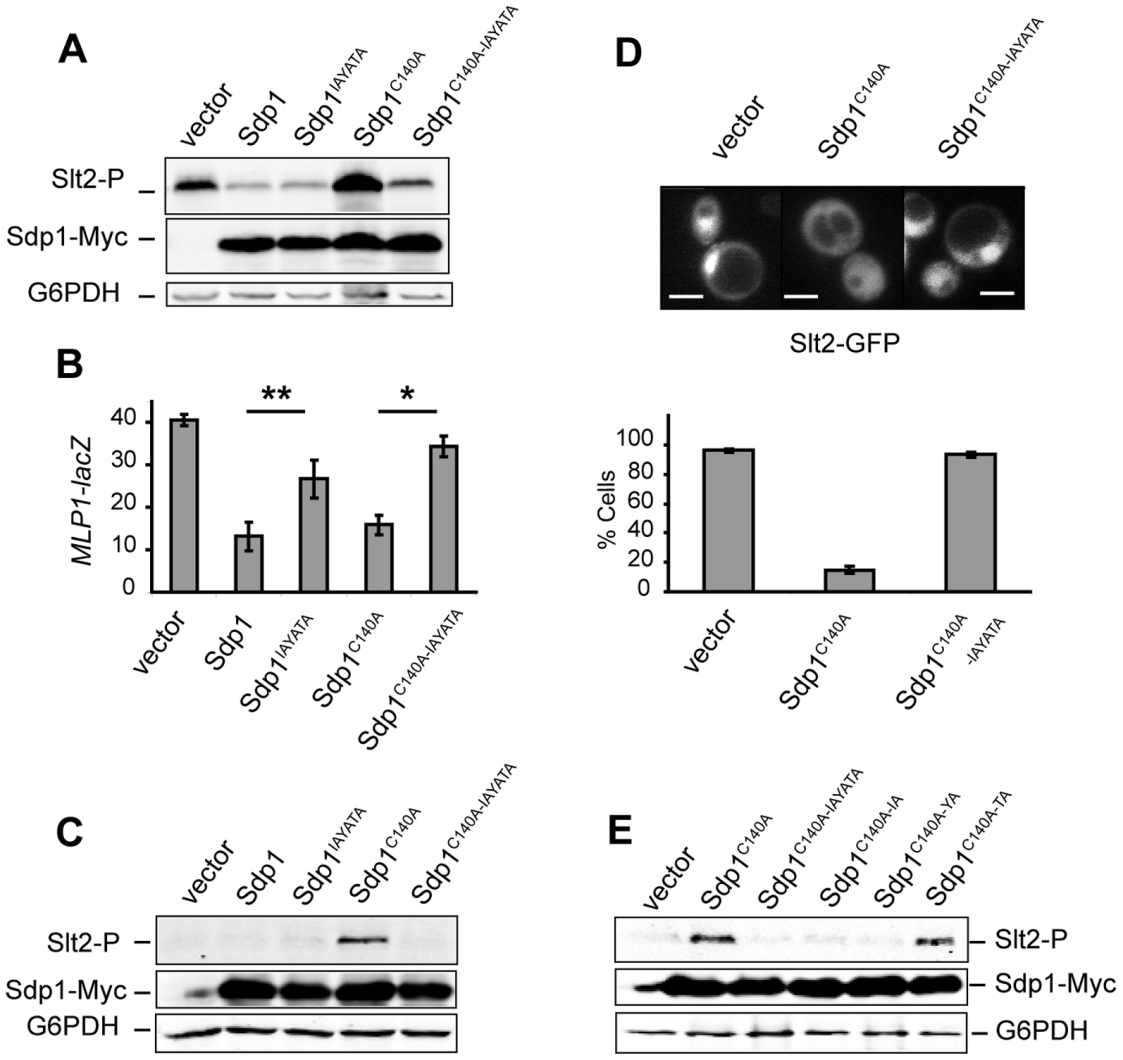
Effect of the lack of the IYT motif of Sdp1 on the CWI pathway. (A) Western blotting analysis of the Y00897 (*gas1*Δ) strain transformed with pYES2 (vector), pYES2SDP1myc (Sdp1), pYES2SDP1^IAYATA^myc (Sdp1^IAYATA^), pYES2SDP1^C140A^ (Sdp1^C140A^) or pYES2SDP1^C140A-IAYATA^ (Sdp1^C140A-IAYATA^) plasmids. Cells were cultured to mid-log phase in raffinose-based selective medium at 24°C, then galactose was added to a final 2% concentration for 4 hours at 24°C, and protein extracts were prepared. Phospho-Slt2, Sdp1-Myc and G6PDH (as a loading control) were detected with anti-phospho-p42/44 (upper panel), anti-Myc (middle panel) and anti-G6PDH (lower panel) antibodies, respectively. (B) Expression of *MLP1-lacZ* was examined by determining β-galactosidase activity in cell extracts of the same transformants as in *A* also carrying the *pMLP1*-LACZ plasmid. Data shown are the average of three independent experiments performed in duplicate. Error bars indicate standard deviations. (C) Western blotting analysis of the wild type YPH499 strain transformed with the same plasmids as in Figure 2A. Cell growth conditions, extract preparation and immunoblot analysis was performed as in Figure 2A. (D) *In vivo* localisation of Slt2-GFP in YPH499 yeast cells carrying the plasmid pRS425-*SLT2*-GFP transformed with pYES2 (vector), pYES2SDP1^C140A^ (Sdp1^C140A^) or pYES2SDP1^C140A-IAYATA^ (Sdp1^C140A-IAYATA^) plasmids. Cells were grown to mid-log phase in raffinose-based selective medium at 24°C, and then galactose was added to a final 2% concentration for 4 hours at 24°C. Fluorescence microscopy photographs showing the cellular distribution of the fluorescent Slt2-GFP were taken. Bars, 5 µm. The percentage of cells showing nuclear accumulation of Slt2-GFP from the same cultures was determined. Data shown are the average of three independent experiments in which n>100. Error bars indicate standard deviations (E) Western blotting analysis of the WT BY4741 strain transformed with pYES2 (vector), pYES2SDP1^C140A^ (Sdp1^C140A^), pYES2SDP1^C140A-IAYATA^ (Sdp1^C140A-IAYATA^), pYES2SDP1^C140A-IA^ (Sdp1^C140A-IA^), pYES2SDP1^C140A-YA^ (Sdp1^C140A-YA^) or pYES2SDP1^C140A-TA^ (Sdp1^C140A-TA^) plasmids. Cells were cultured and extracts processed as in Figure 3A. Similar results were obtained in three different experiments and selected images correspond to representative blots.

It is known that Slt2 concentrates in the nucleus, although it is also located at the cytoplasm [13], [29]. As shown in Figure 3D, the overexpression of inactive Sdp1^C140A^ significantly reduced the nuclear localisation of this MAPK, suggesting that the trapping effect prevents the translocation of Slt2 to the nucleus. The IYT motif was necessary for Sdp1^C140A^ to promote this effect, as cells overexpressing Sdp1^C140A-IAYATA^ showed nuclear accumulation of Slt2 (Figure 3D). Collectively these experiments support the notion that this motif indeed mediates the interaction between Sdp1 and Slt2.

In order to determine the relevance of each residue within the IYT motif, we generated versions of Sdp1^C140A^ in which Ile^3^, Tyr^4^ and Thr^5^ were individually changed to Ala and the effect on Slt2 trapping was analysed. As can be observed in Figure 3E, mutation of any of the two first amino acids in the motif led to a failure of inactive Sdp1 to trap Slt2. However, mutation of the third residue did not preclude trapping of the MAPK, indicating that binding is not disrupted by this mutation. In summary, only the invariant amino acids, namely Ile and Tyr (Figure 1C), are indispensable for this motif to act as a MAPK docking site.

### Mutation of the IYT motif of Msg5 reduces Slt2 but not Fus3 trapping

**Figure 4:**
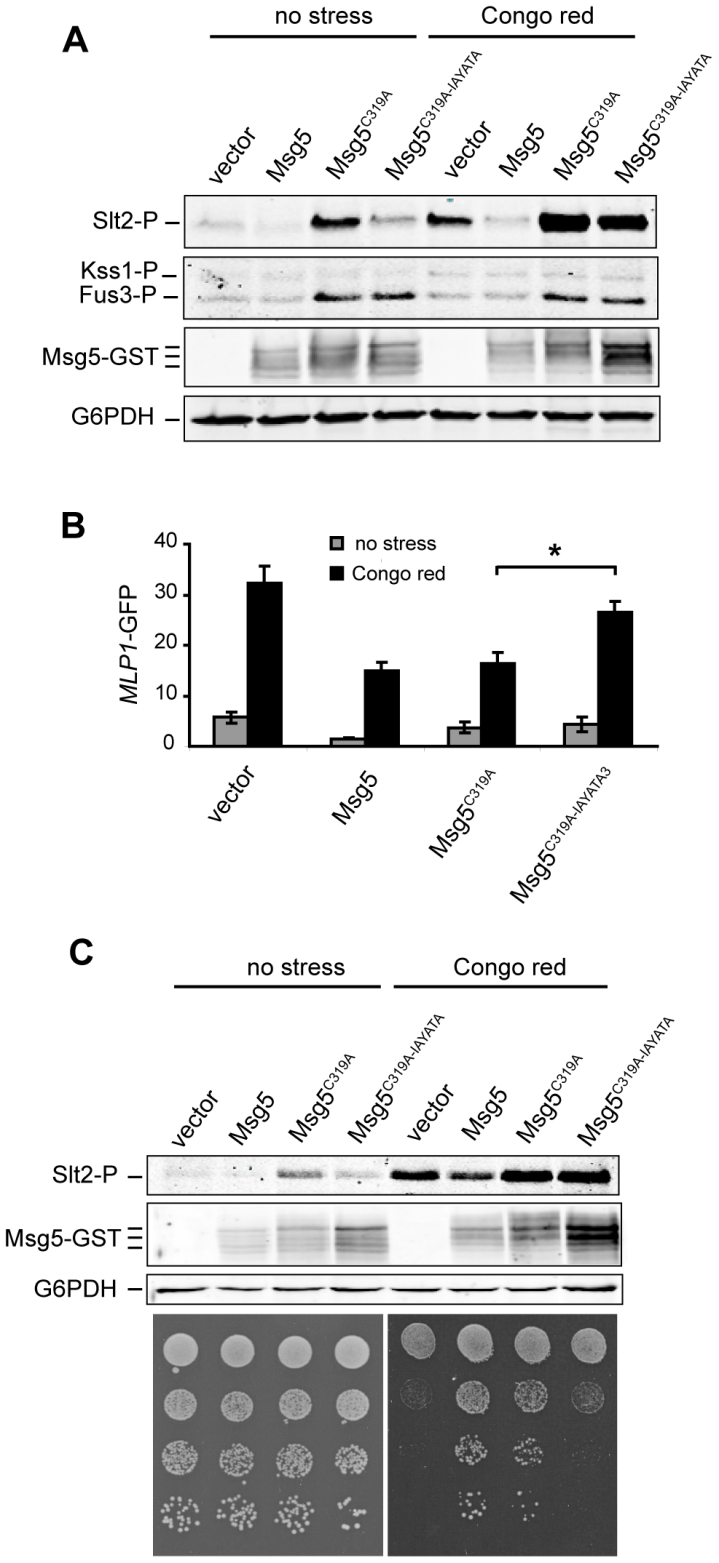
Effect of the lack of the IYT motif of inactive Msg5 on MAPK trapping. (A) Western blotting analysis of the wild type strain YPH499 bearing YEp352GST (vector), YEp352MSG5GST (Msg5), YEp352MSG5^C319A^GST (Msg5^C319A^) or YEp352MSG5^C319A-IAYATA^GST (Msg5^C319A-IAYATA^) plasmids. Cells were grown to mid-log phase at 24°C and then aliquots were treated or not with Congo red (30 µg/ml) for 180 min. Protein extracts were prepared and phosphorylated MAPKs (Slt2, Kss1 and Fus3), Msg5-GST and G6PDH (as a loading control) were detected with anti-phospho-p42/44 (two upper panels), anti-GST antibody (middle panel) and anti-G6PDH antibodies (lower panel), respectively. (B) Expression of *MLP1-GFP* was examined by determining GFP signal in the same transformants (additionally carrying *pMLP1*-*GFP* plasmid) and growth conditions as in Figure 4A. Data shown are the average of three independent experiments performed in duplicate. Error bars indicate standard deviations. (C) Upper panels: Western blotting analysis of the *msg5Δ* mutant strain DD1-2D, carrying the same plasmids as in Figure 4A. Lower panels: sensitivity to Congo red of the same transformants as above. Cells were grown in liquid selective medium at 24°C, and a 10-fold dilution series of this culture was spotted onto YPD agar medium in the absence (no stress) or presence of Congo Red (75 µg/ml) and incubated for 2 days at 24°C. Similar results were obtained in three different experiments and selected images correspond to representative blots.

We next studied whether mutation of the IYT motif also reduced the substrate trapping ability of inactive Msg5 on Slt2. To this end, we analysed, as above, the amount of phosphorylated Slt2 and *MLP1*-driven *lacZ* expression in wild type cells overexpressing Msg5^C319A^, which carries a inactivating mutation at the catalytic site [17], and Msg5^C319A-IAYATA^ that additionally lacks the IYT motif. As shown in Figure 4*A*, the overexpression of Msg5^C319A^ from a multicopy plasmid under its own promoter resulted in increased levels of phosphorylated Slt2 relative to cells carrying the empty vector or overexpressing wild type Msg5, both in the absence or the presence of Congo red, which is an agent that affects the cell wall, therefore leading to CWI pathway activation [30]. The accumulation of phosphorylated Slt2, due to the substrate trapping activity of the inactive Msg5 version, was significantly reduced in cells bearing Msg5^C319A-IAYATA^, suggesting a reduced interaction of this version of Msg5 with Slt2. Remarkably, Msg5^C319A^ also led to Fus3 trapping (Figure 4A), but the accumulation of phosphorylated Fus3 was not reduced by removal of the IYT site within Msg5^C319A^. This result confirms that this site does not mediate the interaction of Msg5 with the mating MAPK, as previously reported [16]. Similarly to that occurring in Sdp1^C140A^-expressing cells, the increased Slt2 phosphorylation promoted by Msg5^C319A^ does not lead to an increased pathway output, but, on the contrary, to a reduction of *MLP1* transcription compared to control cells, as revealed by β-galactosidase activity assays (Figure 4B). Removal of the IYT site in the inactive Msg5 led to an increase in Rlm1 transcriptional activity, indicating a significant reduction of the trapping effect.

We further analysed the functionality of Msg5 versions by monitoring their effect on the Congo red sensitivity displayed by an *msg5*Δ strain. This phenotype is indicative of an affected cell wall, likely derived from an altered Slt2-mediated response in this mutant [12]. As expected, expression of wild type Msg5 complemented the Congo red-sensitivity phenotype of cells lacking this phosphatase (Figure 4C). Of interest, the inactive Msg5^C1319A^ version also rescued the Congo red sensitivity of the *msg5*Δ strain, probably owing to its substrate trapping effect, which leads to a diminished signalling output by antagonising the interaction of Slt2 with Rlm1. In contrast, the *msg5*Δ phenotype could not be rescued by Msg5^C319A-IAYATA^, suggesting the inability of this version to reduce the CWI pathway response due to a lack of the docking site in the trapping inactive mutant.

### The IYT motif mediates binding of MKP Pmp1 to Pmk1 MAPK in fission yeast

**Figure 5:**
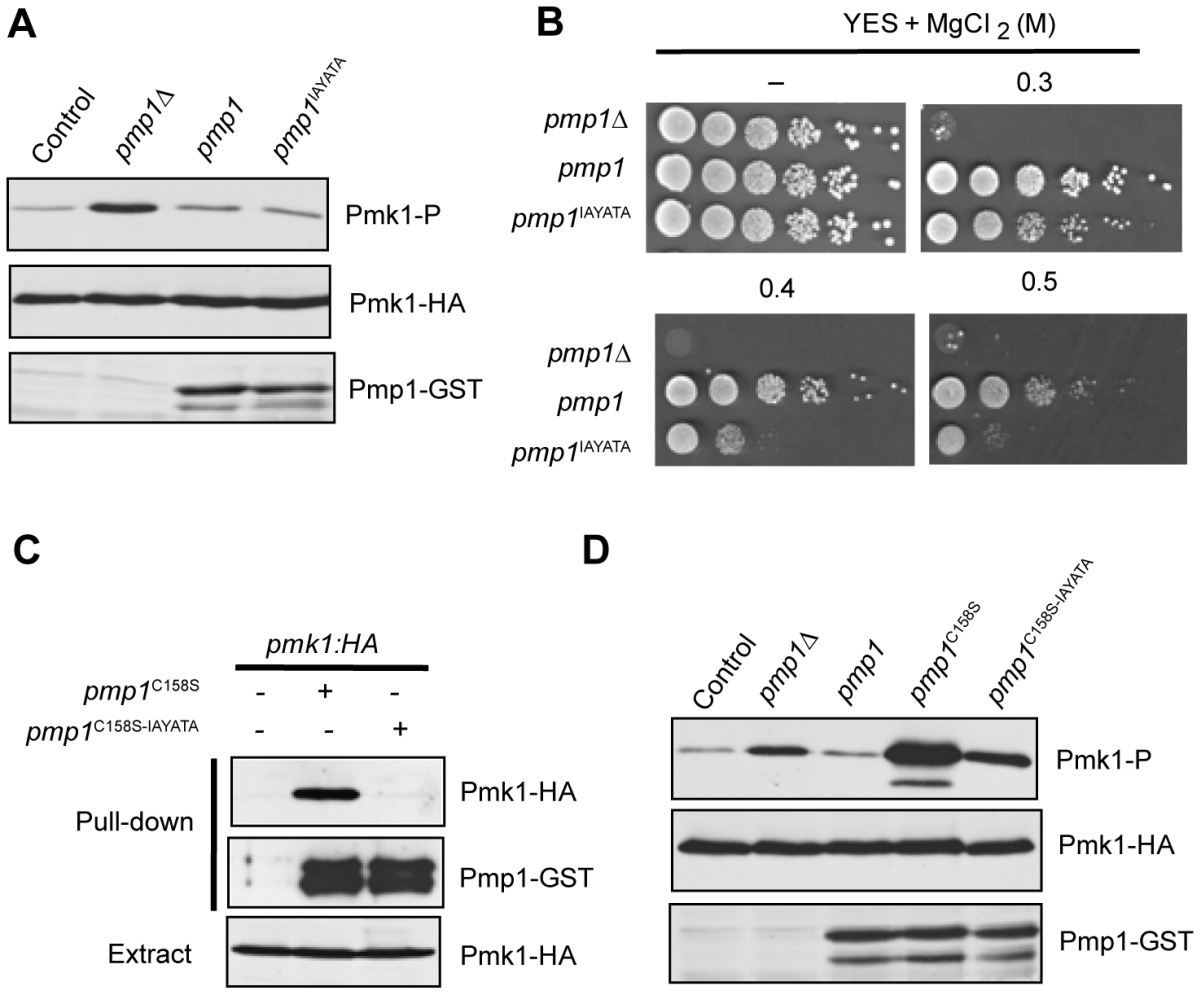
Conserved IYT motif participates in binding of Pmp1 phosphatase to Pmk1 MAPK in *S. pombe*. (A) Strains MI200 (Pmk1-HA6H; Control), MI212 (*pmp1Δ*, Pmk1-HA6H), DP400 (*pmp1Δ, pmp1-GST, pmk1-HA6H*), and DP420 (*pmp1Δ, pmp1(I10A Y11A T12A)-GST, pmk1-HA6H*) were grown in YES medium to early-log phase, extracts were prepared and Pmk1-HA6H was purified by affinity chromatography. Phosphorylated Pmk1 was detected by immunoblotting with anti-phosho-p42/44 antibody (upper panel), total Pmk1 with anti-HA antibody as loading control (middle panel), and total Pmp1 with anti-GST antibody. (B) Ten-fold dilution series of cultures from strains MI212, DP400 and DP420 were spotted on YES plates or YES plates supplemented with either 0.3, 0.4 and 0.5 M MgCl_2_, and incubated for 3 days at 28°C. (C) Strains MI200 (control, lane 1), DP410 (*pmp1Δ, pmp1(C158S)-GST, pmk1-HA6H*; lane 2) and DP430 (*pmp1Δ, pmp1(I10A Y11A T12A C158S)-GST, pmk1-HA6H*; lane 3) were grown in YES medium to early-log phase, extracts were prepared and Pmp1-GST fusions were recovered after incubation with glutathione(GSH)-agarose beads. After extensive washes, the bound proteins were detected by immunoblotting with anti-HA (upper panel) and anti-GST antibodies (middle panel). Total extracts from the above strains were probed with anti-HA antibody as a loading control (lower panel). (D) Strains MI200 (Control), MI212 (*pmp1Δ*), DP400 (*pmp1-GST*), DP410 (*pmp1(C158S)-GST*) and DP430 (*pmp1(I10A Y11A T12A C158S)-GST*) were grown in YES medium to early-log phase, extracts were prepared and Pmk1-HA6H was purified by affinity chromatography. Immunodetection was performed as in A. Similar results were obtained in three different experiments and selected images correspond to representative blots.

We next investigated the functional relevance of the IYT motif in the interaction between the MKP Pmp1 and the MAPK Pmk1 of the CWI pathway in *S. pombe* [15],[31]. The N-terminal end of Pmp1 contains a perfectly conserved IYT motif at positions 10–12 (Figure 1A). As previously reported [15], basal Pmk1 phosphorylation of a mutant lacking Pmp1 phosphatase is increased in comparison to control cells (Figure 5A). The Pmk1 hyperactivation displayed by this mutant has a dramatic effect on chloride homeostasis in fission yeast and consequently leads to strong sensitivity to this anion (Figure 5B; [15]). Expression in *pmp1Δ* cells of genomic C-terminal GST-tagged versions of either wild type Pmp1 or the mutant Pmp1^IAYATA^ version, in which the I^10^Y^11^T^12^ motif was substituted by three consecutive alanines, reduced Pmk1 phosphorylation to a similar extent (Figure 5A). However, Pmp1^IAYATA^ was shown to be less effective than wild type Pmp1 with regard to suppressing the chloride sensitivity phenotype of *pmp1Δ* (Figure 5B), indicating that this motif is biologically relevant for Pmp1 function. Indeed, co-purification experiments performed with strains expressing C-terminal epitope tagged genomic copies of Pmp1 and Pmk1 to GST and HA6H, respectively, revealed the importance of the IYT motif of Pmp1 for the interaction with Pmk1. As observed in Figure 5C, the *in vivo* binding of a catalytically inactive version of Pmp1 (Pmp1^C158S^) to Pmk1 resulted in abrogated function when the inactive phosphatase version also lacked the IYT motif (Pmp1^C158S-IAYATA^). Similar to the results obtained with both Msg5 and Sdp1 in budding yeast, expression of inactive Pmp1^C158S^ induced a strong raise in the level of phosphorylated Pmk1 (Figure 5E). Notably, this high Pmk1 phosphorylation was greatly reduced in cells expressing Pmp1^C158S-IAYATA^ (Figure 5E), confirming the role of the IYT motif of Pmp1 in binding and trapping Pmk1.

## Discussion

It is well accepted that the specificity and efficiency of action of MAPK phosphatases (MKPs) on their cognate MAPKs are controlled by docking interactions. We found in a previous work that the interaction of the *S. cerevisiae* dual-specificity phosphatase Msg5 with MAPKs of different signalling pathways, namely mating and CWI MAPKs, was mediated by distinct docking mechanisms. Particularly, this MPK uses a conventional D-domain to interact with the CD domain of Fus3 and Kss1, whereas a novel motif (IYT) was responsible for binding to Slt2 and its paralogue Mlp1 in a CD-independent manner [16]. Here, we present evidence that the IYT motif acts as an efficient and specific docking site on other fungal Msg5-like MKPs for binding to their target CWI MAPKs. Several results obtained in the present study support this conclusion. First, removal of the IYT motif in both Sdp1 from *S. cerevisiae* and Pmp1 from *S. pombe* precludes interaction with Slt2 and Mlp1, and with Pmk1, respectively. Second, substrate-trapping inactive versions of ScMsg5, ScSdp1 and SpPmp1 lacking this motif have a reduced ability to trap their target CWI MAPKs. Third, whereas inactive Sdp1 prevents Slt2 from concentrating in the yeast nucleus, this Sdp1 version lacking the IYT motif does not significantly alter Slt2 localisation. Fourth, the IYT sequence is present in the non-catalytic region of many fungal Msg5 orthologues. Although most of them belong to genera close to *Saccharomyces*, this motif is also born by the MKP Pmp1 of the evolutionarily distant yeast *S. pombe*. This suggests that this docking motif was present in ancestral MKPs and has probably been maintained through evolution in some MKPs for a specific purpose, namely the regulation of CWI MAPKs.

In contrast to other fungal species, *S. cerevisiae* possesses two MKPs [11], Msg5 and Sdp1, likely generated from the ancient whole genome duplication that occurred in this yeast [32]. However, whereas Msg5 is able to inactivate mating and CWI MAPKs, Sdp1 displays a selective action on Slt2, reflecting functional divergence along evolution. The presence in Msg5 of a large N-terminal non-catalytic region carrying both the characteristic D-domain of most MKPs and the IYT docking motif explains why this phosphatase regulates both types of MAPKs. Pmk1 is the only reported MAPK regulated by the *S. pombe* MKP Pmp1. The short N-terminal regulatory region containing an IYT motif but lacking the typical D-domain in Pmp1 is the most likely reason for its exclusive targeting to the CWI-MAPK Pmk1.

Although the crystal structure of Sdp1 has been previously determined [33], it corresponds to a truncated version lacking 16 residues from the amino-terminus, and thus the IYT motif. Therefore, the structural basis underlying the binding mechanism involving this docking site cannot be readily predicted. Analysis of the region upstream the IYT motif in fungal Msg5 orthologues revealed other residues that were relatively conserved, such as a negatively-charged amino acid at position −3 and two basic residues at position −5. However, the absence of this region in Sdp1 together with the reported dispensability of the two arginines at positions 96 and 97 in *S. cerevisiae* Msg5 for binding to both Slt2 and Mlp1 [16] suggest that interaction through the IYT motif does not require these previous residues. Moreover, mutational analysis of each of the residues composing the IYT motif in Sdp1 showed that only the invariant amino acids Ile and Tyr are essential for binding. Of interest, we have found that atypical human DSPs DSP18 and DSP21, which lack any known specific MAPK targeting motif, carry an IYE sequence at their unique C-terminal extension [34]. The fact that the third residue seems to be dispensable in fungal MKPs for MAPK binding suggests that this IYE sequence could be also acting as a docking site in these human DSPs. This opens up the possibility that this motif has been shaped through evolution in these atypical phosphatases to mediate interactions with target MAPKs in higher eukaryotes.

Mutations that reduce the catalytic activity of protein phosphatases without affecting their substrate affinity have been named “substrate-trapping”, due to their ability to form a stable enzyme-substrate complex [28]. In the case of MKPs, the formation of such complexes with the MAPK may antagonise binding between the MAPK and its downstream substrates impeding signalling transmission. This is indeed what we observed when catalytically inactive versions of Msg5, Sdp1 and Pmp1, mutated in the conserved essential Cys at the active site, are overexpressed in yeast cells. The lack of phosphatase activity of these substrate-trapping mutant versions led to phosphorylation of the corresponding MAPK in the complexes. However, these phosphorylated and therefore active MAPKs are paradoxically unable to activate their targets. Elimination of the IYT docking motif in such inactive MKPs released the partner MAPK, namely Slt2 in *S. cerevisiae* or Pmk1 in *S. pombe*, from the trapping complex, allowing signal propagation. In view of these results, the analysis of MAPK phosphorylation and specific transcriptional induction upon overexpression of a catalytically inactive MKP is a powerful approach for identification of target MAPKs of such MKP. This strategy can be especially useful for MKPs whose deletion or overexpression does not give rise to an evident phenotype, like Sdp1. Msg5 has proven to be a more potent phosphatase than Sdp1, controlling Slt2 phosphorylation both under basal and stimulating conditions [18], [35], whereas Sdp1 seems to dephosphorylate Slt2 only upon heat stress activation [13]. However, we could not detect increased phosphorylation of Slt2 at high temperature in any *sdp1Δ* mutant strain analysed (data not shown). Moreover, recent large-scale analysis in *S. cerevisiae* comparing the impact of every single phosphatase gene deletion on either the global expression profile or the phosphoproteome also showed that the absence of Sdp1 affects gene transcription and protein phosphorylation to a much lesser extent tan Msg5 [36], [37]. This is consistent with the lack of an apparent phenotype of *sdp1Δ* mutants under cell wall stress conditions (data not shown), while an *msg5Δ* mutant is hypersensitive to this stress [12]. The fact that the inactive Msg5 trapping mutant, which interferes with signalling from Slt2 to downstream effectors, suppressed the Congo red hypersensitivity of *msg5Δ* cells supports the idea that this phenotype is due to an up-regulated CWI pathway response.

In summary, our work provides evidence on the conservation of an interaction motif in MKPs for selective binding to CWI MAPKs. Furthermore, we unravel the essential residues within this MKP motif for binding to MAPKs. Since various MAPKs can be regulated by the same MKP and, conversely, several MKPs are able to dephosphorylate a particular MAPK, the existence of different mechanisms mediating binding of these proteins ensures accurate signal transduction. Our findings reveal the high flexibility of these proteins to differentially regulate structurally similar MAPKs.

## References

[1] Cargnello M, Roux PP (2011) Activation and function of the MAPKs and their substrates, the MAPK-activated protein kinases. Microbiol Mol Biol Rev 75: 50–83. 21372320 10.1128/MMBR.00031-10 PMC3063353

[2] Martin H, Flandez M, Nombela C, Molina M (2005) Protein phosphatases in MAPK signalling: we keep learning from yeast. Mol Microbiol 58: 6–16. 16164545 10.1111/j.1365-2958.2005.04822.x

[3] Caunt CJ, Keyse SM (2013) Dual-specificity MAP kinase phosphatases (MKPs): shaping the outcome of MAP kinase signalling. FEBS J 280: 489–504. 22812510 10.1111/j.1742-4658.2012.08716.x PMC3594966

[4] Tanoue T, Nishida E (2002) Docking interactions in the mitogen-activated protein kinase cascades. Pharmacol Pharmacol Ther 93: 193–202. 12191611 10.1016/s0163-7258(02)00188-2

[5] Bardwell L, Shah K (2006) Analysis of mitogen-activated protein kinase activation and interactions with regulators and substrates. Methods 40: 213–223. 16884917 10.1016/j.ymeth.2006.06.008 PMC3017500

[6] Akella R, Moon TM, Goldsmith EJ (2008) Unique MAP Kinase binding sites. Biochim Biophys Acta 1784: 48–55. 18068683 10.1016/j.bbapap.2007.09.016 PMC2266891

[7] Lin YW, Yang JL (2006) Cooperation of ERK and SCFSkp2 for MKP-1 destruction provides a positive feedback regulation of proliferating signaling. J Biol Chem 281: 915–926. 16286470 10.1074/jbc.M508720200

[8] Lee T, Hoofnagle AN, Kabuyama Y, Stroud J, Min X, et al (2004) Docking motif interactions in MAP kinases revealed by hydrogen exchange mass spectrometry. Mol Cell 14: 43–55. 15068802 10.1016/s1097-2765(04)00161-3

[9] Chen RE, Thorner J (2007) Function and regulation in MAPK signaling pathways: Lessons learned from the yeast *Saccharomyces cerevisiae* . Biochim Biophys Acta 1773: 1311–1340. 17604854 10.1016/j.bbamcr.2007.05.003 PMC2031910

[10] Levin DE (2011) Regulation of cell wall biogenesis in *Saccharomyces cerevisiae*: the cell wall integrity signaling pathway. Genetics 189: 1145–1175. 22174182 10.1534/genetics.111.128264 PMC3241422

[11] Rispail N, Soanes DM, Ant C, Czajkowski R, Grunler A, et al (2009) Comparative genomics of MAP kinase and calcium-calcineurin signalling components in plant and human pathogenic fungi. Fungal Genet Biol 46: 287–298. 19570501 10.1016/j.fgb.2009.01.002

[12] Flandez M, Cosano IC, Nombela C, Martin H, Molina M (2004) Reciprocal regulation between Slt2 MAPK and isoforms of Msg5 dual-specificity protein phosphatase modulates the yeast cell integrity pathway. J Biol Chem 279: 11027–11034. 14703512 10.1074/jbc.M306412200

[13] Hahn JS, Thiele DJ (2002) Regulation of the *Saccharomyces cerevisiae* Slt2 kinase pathway by the stress-inducible Sdp1 dual specificity phosphatase. J Biol Chem 277: 21278–21284. 11923319 10.1074/jbc.M202557200

[14] Collister M, Didmon MP, MacIsaac F, Stark MJ, MacDonald NQ, et al (2002) YIL113w encodes a functional dual-specificity protein phosphatase which specifically interacts with and inactivates the Slt2/Mpk1p MAP kinase in *S. cerevisiae* . FEBS Lett 527: 186–192. 12220658 10.1016/s0014-5793(02)03220-9

[15] Sugiura R, Toda T, Shuntoh H, Yanagida M, Kuno T (1998) pmp1+, a suppressor of calcineurin deficiency, encodes a novel MAP kinase phosphatase in fission yeast. EMBO J 17: 140–148. 9427748 10.1093/emboj/17.1.140 PMC1170365

[16] Palacios L, Dickinson RJ, Sacristan-Reviriego A, Didmon MP, Marin MJ, et al (2011) Distinct docking mechanisms mediate interactions between the Msg5 phosphatase and mating or cell integrity mitogen-activated protein kinases (MAPKs) in *Saccharomyces cerevisia*e. J Biol Chem 286: 42037–42050. 22006927 10.1074/jbc.M111.286948 PMC3234975

[17] Doi K, Gartner A, Ammerer G, Errede B, Shinkawa H, et al (1994) MSG5, a novel protein phosphatase promotes adaptation to pheromone response in *S. cerevisiae* . EMBO J 13: 61–70. 8306972 10.1002/j.1460-2075.1994.tb06235.x PMC394779

[18] Martin H, Rodriguez-Pachon JM, Ruiz C, Nombela C, Molina M (2000) Regulatory mechanisms for modulation of signaling through the cell integrity Slt2-mediated pathway in *Saccharomyces cerevisiae* . J Biol Chem 275: 1511–1519. 10625705 10.1074/jbc.275.2.1511

[19] Madrid M, Soto T, Khong HK, Franco A, Vicente J, et al (2006) Stress-induced response, localization, and regulation of the Pmk1 cell integrity pathway in *Schizosaccharomyces pombe* . J Biol Chem 281: 2033–2043. 16291757 10.1074/jbc.M506467200

[20] Madrid M, Nunez A, Soto T, Vicente-Soler J, Gacto M, et al (2007) Stress-activated protein kinase-mediated down-regulation of the cell integrity pathway mitogen-activated protein kinase Pmk1p by protein phosphatases. Mol Biol Cell 18: 4405–4419. 17761528 10.1091/mbc.E07-05-0484 PMC2043569

[21] Mitchell DA, Marshall TK, Deschenes RJ (1993) Vectors for the inducible overexpression of glutathione S-transferase fusion proteins in yeast. Yeast 9: 715–722. 8368005 10.1002/yea.320090705

[22] Sharrocks AD (1994) A T7 expression vector for producing N- and C-terminal fusion proteins with glutathione S-transferase. Gene 138: 105–108. 8125285 10.1016/0378-1119(94)90789-7

[23] Hill JE, Myers AM, Koerner TJ, Tzagoloff A (1986) Yeast/E. coli shuttle vectors with multiple unique restriction sites. Yeast 2: 163–167. 3333305 10.1002/yea.320020304

[24] Rodriguez-Pena JM, Diez-Muniz S, Nombela C, Arroyo J (2008) A yeast strain biosensor to detect cell wall-perturbing agents. J Biotechnol 133: 311–317. 18055054 10.1016/j.jbiotec.2007.10.006

[25] Garcia R, Rodriguez-Pena JM, Bermejo C, Nombela C, Arroyo J (2009) The high osmotic response and cell wall integrity pathways cooperate to regulate transcriptional responses to zymolyase-induced cell wall stress in *Saccharomyces cerevisiae* . J Biol Chem 284: 10901–10911. 19234305 10.1074/jbc.M808693200 PMC2667776

[26] Guarente L (1983) Yeast promoters and lacZ fusions designed to study expression of cloned genes in yeast. Methods Enzymol 101: 181–191. 6310321 10.1016/0076-6879(83)01013-7

[27] de Nobel H, Ruiz C, Martin H, Morris W, Brul S, et al (2000) Cell wall perturbation in yeast results in dual phosphorylation of the Slt2/Mpk1 MAP kinase and in an Slt2-mediated increase in FKS2-lacZ expression, glucanase resistance and thermotolerance. Microbiology 146: 2121–2132. 10974100 10.1099/00221287-146-9-2121

[28] Tonks NK, Neel BG (1996) From form to function: signaling by protein tyrosine phosphatases. Cell 87: 365–368. 8898190 10.1016/s0092-8674(00)81357-4

[29] Kamada Y, Jung US, Piotrowski J, Levin DE (1995) The protein kinase C-activated MAP kinase pathway of *Saccharomyces cerevisiae* mediates a novel aspect of the heat shock response. Genes Dev 9: 1559–1571. 7628692 10.1101/gad.9.13.1559

[30] Marin MJ, Flandez M, Bermejo C, Arroyo J, Martin H, et al (2009) Different modulation of the outputs of yeast MAPK-mediated pathways by distinct stimuli and isoforms of the dual-specificity phosphatase Msg5. Mol Genet Genomics 281: 345–359. 19123063 10.1007/s00438-008-0415-5

[31] Toda T, Dhut S, Superti-Furga G, Gotoh Y, Nishida E, et al (1996) The fission yeast pmk1+ gene encodes a novel mitogen-activated protein kinase homolog which regulates cell integrity and functions coordinately with the protein kinase C pathway. Mol Cell Biol 16: 6752–6764. 8943330 10.1128/mcb.16.12.6752 PMC231678

[32] Kellis M, Birren BW, Lander ES (2004) Proof and evolutionary analysis of ancient genome duplication in the yeast *Saccharomyces cerevisiae* . Nature 428: 617–624. 15004568 10.1038/nature02424

[33] Fox GC, Shafiq M, Briggs DC, Knowles PP, Collister M, et al (2007) Redox-mediated substrate recognition by Sdp1 defines a new group of tyrosine phosphatases. Nature 447: 487–492. 17495930 10.1038/nature05804

[34] Jeong DG, Cho YH, Yoon TS, Kim JH, Son JH, et al (2006) Structure of human DSP18, a member of the dual-specificity protein tyrosine phosphatase family. Acta Crystallogr D Biol Crystallogr 62: 582–588. 16699184 10.1107/S0907444906010109

[35] Harrison JC, Zyla TR, Bardes ES, Lew DJ (2004) Stress-specific activation mechanisms for the “cell integrity” MAPK pathway. J Biol Chem 279: 2616–2622. 14610085 10.1074/jbc.M306110200

[36] van Wageningen WS, Kemmeren P, Lijnzaad P, Margaritis T, Benschop JJ, et al (2010) Functional overlap and regulatory links shape genetic interactions between signaling pathways. Cell 143: 991–1004. 21145464 10.1016/j.cell.2010.11.021 PMC3073509

[37] Bodenmiller B, Wanka S, Kraft C, Urban J, Campbell D, et al (2010) Phosphoproteomic analysis reveals interconnected system-wide responses to perturbations of kinases and phosphatases in yeast. Sci Signal 3: rs4 Available: http://stke.sciencemag.org/cgi/content/full/sigtrans3/153/rs4. Accessed 21 December 2010. 21177495 10.1126/scisignal.2001182 PMC3072779

[38] Bailey TL, Boden M, Buske FA, Frith M, Grant CE, et al (2009) MEME SUITE: tools for motif discovery and searching. Nucleic Acids Res 37: W202–W208. 19458158 10.1093/nar/gkp335 PMC2703892

